# Screening macroalgae for mitigation of enteric methane in vitro

**DOI:** 10.1038/s41598-023-36359-y

**Published:** 2023-06-17

**Authors:** D. E. Wasson, H. Stefenoni, S. F. Cueva, C. Lage, S. E. Räisänen, A. Melgar, M. Fetter, M. Hennessy, K. Narayan, N. Indugu, D. Pitta, C. Yarish, A. N. Hristov

**Affiliations:** 1 Department of Animal Science, The Pennsylvania State University, University Park, PA 16802 USA; 2 Department of Environmental Sciences, Institute of Agricultural Sciences, ETH Zürich, 8092 Zürich, Switzerland; 3 Agricultural Innovation Institute of Panama (IDIAP), 161 Carlos Lara Street, City of Knowledge, 07144 Panama; 4 Department of Clinical Studies, New Bolton Center, School of Veterinary Medicine, University of Pennsylvania, Kennett Square, PA 19348 USA; 5 Department of Ecology and Evolutionary Biology, University of Connecticut, Stamford, CT 06901 USA

**Keywords:** Microbiology, Environmental sciences, Climate-change mitigation

## Abstract

This study investigated the effects of 67 species of macroalgae on methanogenesis and rumen fermentation in vitro. Specimens were analyzed for their effect on ruminal fermentation and microbial community profiles. Incubations were carried out in an automated gas production system for 24-h and macroalgae were tested at 2% (feed dry matter basis) inclusion rate. Methane yield was decreased 99% by *Asparagopsis taxiformis* (AT) when compared with the control. *Colpomenia peregrina* also decreased methane yield 14% compared with control; no other species influenced methane yield. Total gas production was decreased 14 and 10% by AT and *Sargassum horneri* compared with control, respectively. Total volatile fatty acid (VFA) concentration was decreased between 5 and 8% by 3 macroalgae, whereas AT reduced it by 10%. Molar proportion of acetate was decreased 9% by AT, along with an increase in propionate by 14%. *Asparagopsis taxiformis* also increased butyrate and valerate molar proportions by 7 and 24%, respectively, whereas 3 macroalgae species decreased molar proportion of butyrate 3 to 5%. *Vertebrata lanosa* increased ammonia concentration, whereas 3 other species decreased it. Inclusion of AT decreased relative abundance of *Prevotella, Bacteroidales, Firmicutes and Methanobacteriaceae,* whereas *Clostridium, Anaerovibrio* and *Methanobrevibacter* were increased. Specific gene activities for *Methanosphaera stadtmane* and *Methanobrevibacter ruminantium* were decreased by AT inclusion. In this in vitro study, *Asparagopsis taxiformis* was most effective in decreasing methane concentration and yield, but also decreased total gas production and VFA concentration which indicates overall inhibition of ruminal fermentation. No other macroalgae were identified as potential mitigants of enteric methane.

## Introduction

According to the United States Environmental Protection Agency (USEPA), in 2020 agriculture was responsible for 11% of the total greenhouse gas (GHG) emissions in the U.S. (on a CO_2_ equivalent basis, CO_2_e) with approximately 30% of these emissions being enteric methane (CH_4_)^1^. Within the United States, dairy and beef cattle contributed approximately 169 million metric tons (MMT) of CH_4_ (43.6 and 125.3 MMT, respectively) on a CO_2_e basis in 2020 through enteric fermentation^1^. Enteric methanogenesis is a process by which various end-products of anerobic microbial fermentation in the rumen, mainly CO_2_ and H_2_, are metabolized by archaea for energy, creating CH_4_^2^_._ Being a downstream product, the quantity of CH_4_ produced is greatly dependent on the initial fermentation substrate [i.e. overall dry matter intake (DMI) and feed type] and rumen conditions. Beyond feed management and formulation, several diet additives that act as CH_4_ inhibitors have been identified^3–5^. Among these, macroalgae, specifically *Asparagopsis taxiformis* (AT), have been identified as a potential candidate to mitigate enteric CH_4_ emission from livestock.

*Asparagopsis taxiformis* was shown to almost eliminate enteric CH_4_ emission in vitro^6^, whereas in vivo research in both cattle and sheep have reported decreases of 0 to 98%, depending on diet and AT inclusion rates^7–10^. While these results are promising, some studies indicated that DMI and milk yield were negatively affected by AT supplementation^9,10^. Reduced animal performance would decrease the effect of AT on a CH_4_ intensity (CH_4_ g/kg milk or meat produced) basis and would likely limit industry adoption of AT.

To the best of our knowledge, AT is not yet cultivated on a commercial scale and wild harvest cannot be sustainable or meet the demand of the global or even U.S. cattle herd of 92 million animals^11^. It has been estimated that 1% dietary inclusion of AT in U.S. cattle diets alone would require 3 to 3.4 million metric tons of dry AT, which is approximately half of the current global production of all macroalgae^12^. *Asparagopsis taxiformis* is a member of the *Rhodophyta* phylum that is typically found in tropical to warm-temperate waters^13^. This geographic preference needs to be considered when evaluating the carbon footprint of AT as a feed supplement for ruminants^14^. Therefore, finding seaweed species that can be grown in aquaculture or sustainably harvested from local waters could provide an advantage of lower CO_2_e inputs.

The antimethanogenic effect of AT has been attributed to halogenated compounds, specifically bromoform (CHBr_3_), but seaweeds contain numerous bioactive compounds that may also inhibit methanogenesis^15^. For example, terrestrial plant compounds with antimicrobial action also found in macroalgae include: halogenated compounds (other than bromoform), alkaloids, phlorotannins, lipids, large polysaccharides, flavonoids and sulfonated glycans^16,17^. This list is by no means exhaustive but indicates a vast potential for discovery, which to this point has not been fully explored. In this context, a native, high biomass, cultivated seaweed like *Saccharina latissima* could be a better fit for the U.S. livestock feed market than AT, which currently is not cultivated locally or at large scales. Current or potential ability to be widely cultivated, opportunities to be cultivated within integrated multi-trophic aquaculture or nutrient bioextraction systems, potential bioactive compound content, and opportunities for sustainable wild harvest are all factors that should be considered for viable commercial implementation.

Therefore, the objective of this study was to determine the effect of macroalgae species (Supplementary Table 1) found in coastal waters of the U. S. on CH_4_ emission, total gas production, microbial profile, and volatile fatty acid and ammonia concentrations in vitro at 2% substrate dry matter (DM) basis inclusion rate, which would be comparable to in vivo feeding rates. We hypothesized that, apart from AT, there may be other macroalgae that could have a sizable CH_4_ mitigation effect.

## Results and discussion

### Total gas production and composition

In this series of incubations, total gas production (TGP) was measured continuously as a proxy for fermentation activity and to calculate emission of CH_4_ and H_2_. Since methanogenesis in the rumen is a first-order kinetic process^18^ and large datasets of in vivo work have described CH_4_ emission as dependent on substrate availability^19^, TGP and CH_4_ emission were expressed on a mL/g of DM basis (i.e., emission yield). For most treatments, H_2_ concentrations measured ranged from not detectable to < 1 mL/g of DM and, therefore, will not be discussed, except for AT.

**Figure 1:**
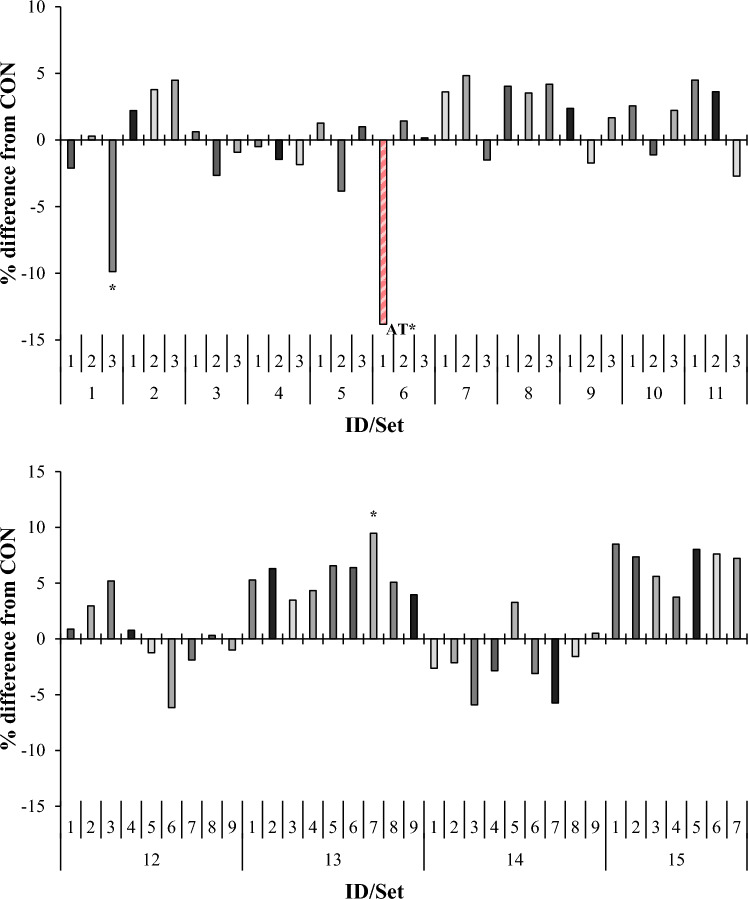
Relative (percent difference, in comparison to set-specific control) effect of macroalgae on 24 h total gas production (mL/g dry matter) in vitro. For set-specific algae identification, see Supplementary Table 1 (ID# within Set). Number of observations used in the statistical analysis: from 26 to 66 (incubation Sets 1 through 15). Mean total gas production for control: 127, 126, 130, 126, 134, 130, 119, 121, 137, 123, 127, 123, 121, 122, and 97 mL/g dry matter, respectively; SEM = 4.1, 2.9, 2.5, 2.4, 5.3, 3.2, 6.2, 3.2, 3.7, 3.3, 4.7, 3.1, 3.5, 4.2, and 3.0, respectively. Means marked with an asterisk differ from the set-specific control (P < 0.05).

**Figure 2:**
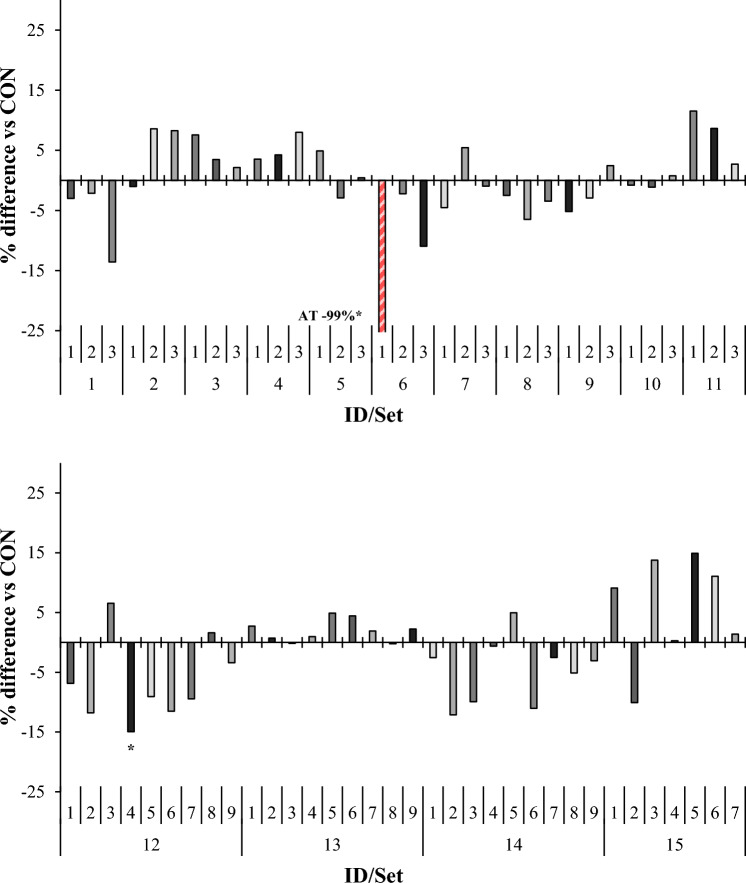
Relative (percent difference, in comparison to set-specific control) effect of macroalgae on 24 h methane yield (mL/g dry matter) in vitro. For set-specific algae identification, see Supplementary Table 1 (ID# within Set). Number of observations used in the statistical analysis: from 26 to 66 (incubation Sets 1 through 15). Mean methane yield for control: 7.97, 9.80, 7.61, 6.13, 8.90, 7.99, 7.71, 7.85, 8.48, 9.06, 7.47, 7.51, 8.01, 7.29, and 5.52 mL/g dry matter, respectively; SEM = 0.815, 0.541, 0.514, 0.367, 1.251, 0.666, 0.856, 0.464, 0.464, 0.617, 0.670, 0.560, 0.446, 0.439, and 0.395, respectively. Means marked with an asterisk differ from the set-specific control (P < 0.05).

*Asparagopsis taxiformis* decreased (*P* < 0.005) 24-h TGP by an average of 14%, when compared with control (Fig. 1). These results are comparable to the 30% reduction in TGP in vitro reported by Kinley et al. (2016) when AT was administered at 2% organic matter (OM) basis^20^. Other studies with AT conducted at the same inclusion level also demonstrated a decrease in TGP^21–23^. In the current study, AT decreased (*P* < 0.001) 24-h CH_4_ yield by 99% compared with control (Fig. 2), again, in agreement with previous studies^20–23^. It is noted that gas and CH_4_ yields from the current experiment may differ numerically from previous in vitro and in vivo studies with AT due to differences in incubation conditions, type of substrate fermented, inclusion rates, and digesta kinetics, in the case of in vivo experiments^9,20,24^. At 24-h, H_2_ emission for AT was drastically increased (*P* < 0.001; from not detectable to 2.19 mL/g DM. This effect is in agreement with other studies, wherein inhibition of methanogenesis resulted in an increase in H_2_ emission^25–29^. *Sargassum horneri* also decreased (*P* < 0.001) TGP 10% when compared with control, but only numerically reduced CH_4_ yield 14%. Comparison of headspace gas samples between *S. horneri* and control revealed no differences in CH_4_ concentration, which indicates that this alga did not inhibit methanogenesis, but decreased CH_4_ emission by inhibiting fermentation and consequently gas production (Supplementary Table 2). Methane emission was also decreased (*P* = 0.05) 13% by *Colpomenia peregrina* without negatively effecting TGP. A related species, *Colpomenia sinuosa*, decreased in vitro CH_4_ production 50% and TGP 10% when included at 20% (OM basis) compared to a decorticated cottonseed meal control^30^. Although direct comparison between these in vitro studies is difficult given the differences in inclusion rates and fermentable substrates [total mixed ration (TMR) vs. Flinders grass hay], this brown algal species may have a methane mitigating effect and should be further investigated. *Pikea californica*, a red seaweed, increased (*P* = 0.03) gas production 9% when compared with control, without a corresponding increase in CH_4_ emission (Supplementary Table 2). Observed differences in gas production could be the result of varying contents of fermentable carbohydrates and/or secondary metabolites which, depending on their mode of action, support or inhibit fermentation^31,32^.

One study^33^ examined a combination of algal species administered at 0.5 to 2.0% of OM. The algae were combined by storms washing them ashore. This combination of macroalgae reduced CH_4_ emission by 12% on average, and up to 16%^33^. Two of the species in this mix (*Chondrus crispus* and *Fucus vesiculosus)* were administered independently (at 0.5% of OM) with no effect on CH_4_ emission, perhaps indicating a complementary effect of including multiple species not seen at an individual level. Similarly, *Chondrus crispus* and *Fucus vesiculosus* had no effect on TGP or CH_4_ emission in the current study. To our knowledge, there are very few studies that investigated the macroalgae species examined in the current experiment at inclusion rates as low as 2% of substrate DM, making comparisons difficult. In many cases algae were tested at a higher dose (2 to 31% of DM) than in the present study^32^. Attempting to draw conclusions about CH_4_ inhibition in lactating cattle at these higher inclusion rates could prove problematic when advanced to in vivo testing stages. Several studies feeding *Rhodophyta* to lactating dairy cows reported decreases in DMI of 3 to 38%, potentially from an aversion to glutamic acid^9,34,35^. Additionally, Roque et al., (2021) reported a decrease in DMI when AT was fed to beef cattle at 0.5% of OM^8^. Therefore, in vitro screening of macroalgae species for enteric CH_4_ mitigation in cattle should be done at lower levels (≤ 2% DM) that will be practical and least likely to hinder palatability of the diet in vivo.

### Volatile fatty acids

**Figure 3:**
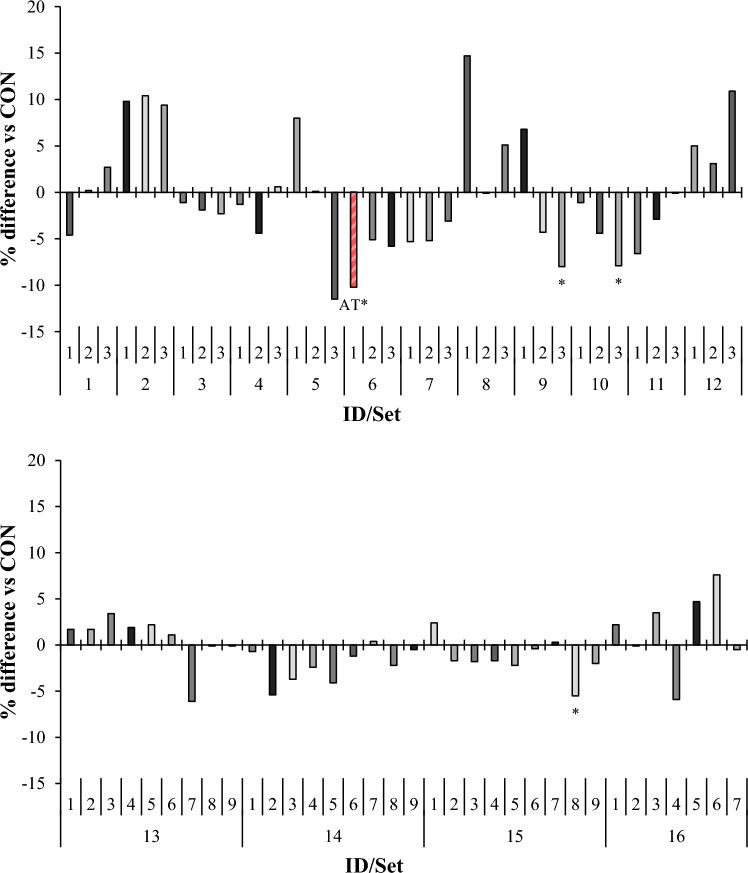
Relative (percent difference, in comparison to set-specific control) effect of macroalgae on total VFA concentration (m*M*) in vitro. For set-specific algae identification, see Supplementary Table 1 (ID# within Set). Number of observations used in the statistical analysis: from 26 to 66 (incubation Sets 1 through 15). Mean Total VFA production for control: 52.1, 75.2, 63.7, 64.6, 59.3, 62.0, 47.5, 59.5, 65.0, 50.4, 59.1, 49.8, 49.6, 49.0, and 46.9 *u*mol/mL, respectively; SEM = 1.43, 3.87, 2.79, 2.45, 4.19, 2.25, 4.09, 2.09, 3.61, 4.37, 3.92, 2.73, 1.37, 0.97, and 2.53, respectively. Means marked with an asterisk differ from the set-specific control (*P* < 0.05).

**Figure 4:**
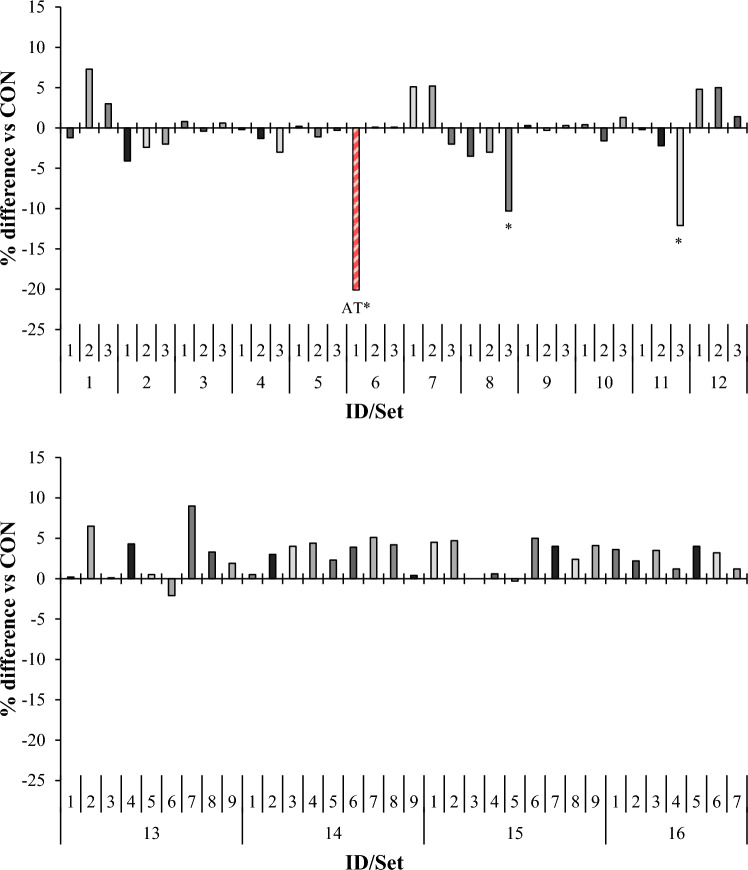
Relative (percent difference, in comparison to set-specific control) effect of macroalgae on acetate:propionate ratio in vitro. For set-specific algae identification, see Supplementary Table 1 (ID# within Set). Number of observations used in the statistical analysis: from 26 to 66 (incubation Sets 1 through 15). Mean acetate:propionate ratio for control: 1.72, 1.82, 2.34, 1.90, 2.06, 1.92, 2.34, 2.63, 2.63, 2.72, 2.38, 2.54, 2.45, 2.61, and 2.25 respectively; SEM = 0.055, 0.085, 0.202, 0.331, 0.427, 0.307, 0.320, 0.085, 0.556, 0.195, 0.943, 1.041, 0.094, and 0.057, respectively. Means marked with an asterisk differ from the set-specific control (*P* < 0.05).

Compared to set-specific controls, total volatile fatty acid (VFA) concentration was decreased (*P* < 0.05) by AT, *Fucus evanescens*, *Ulva intestinalis*, and *Sarcodiotheca gaudichaudii* by 10, 8, 8, and 5%, respectively (Fig. 3). Despite decreasing total VFA concentration, apart from AT, none of these species decreased TGP as would be expected with inhibited fermentation. Additionally, AT decreased (*P* < 0.001) molar proportion of acetate by 9%, increased (*P* < 0.001) propionate by 14%, and subsequently decreased (*P* < 0.001) acetate:propionate ratio by 20% (Fig. 4). The results for AT agree with findings in previous studies at this inclusion level^20,21^. Interestingly, a similar study found no effect of AT on total VFA concentration but reported similar shifts in acetate and propionate molar proportions^22^. Similarly, in vivo supplementation of AT (0.5% DM) decreased total VFA and acetate concentrations (11 and 7%, respectively), and increased propionate molar proportion 8% in dairy cows^9^. In the current study, *Laminaria farlowii* and *Ulva* spp*.* both increased (*P* < 0.05) molar proportion of propionate by 9%, leading to a corresponding decrease (*P* ≤ 0.008) in acetate:propionate ratio of 10 and 12%, respectively.

In the current study, AT increased (*P* < 0.001) the molar proportion of butyrate by 7% when compared with control. *Fucus evanescens* decreased (*P* = 0.04) molar proportion of butyrate by 3%. Isovalerate proportion was increased (*P* = 0.03) 6 to 9% by *Fucus evanescens*, *Ulva intestinalis*, and *Sarcodiotheca gaudichaudii*. Valerate was only affected by AT, which increased (*P* < 0.001) its molar proportion by 24%.

Inhibition of methanogenesis leads to an increase of H_2_ concentration in ruminal fluid and headspace, or the reaction vessel in in vitro systems, as dissolved H_2_ normally consumed in the reduction of CO_2_ by archaea will partially accumulate^36,37^. While not a direct relationship, increased H_2_ concentrations within the headspace is correlated with higher dissolved H_2_ concentrations in ruminal fluid^38–40^. Due to the simplicity of sampling headspace gas, the sensitivity required to analyze low (0.1–50 µM) dissolved H_2_ concentrations, and the low number of studies which report dissolved H_2_, headspace concentrations were used as a proxy for dissolved H_2_ concentrations in the current study. With higher dissolved H_2_ concentrations, production of more H_2_ through fermentation to acetate, becomes energetically unfavorable which results in increased formation of propionate and in some cases butyrate, which act as H^+^ sinks^37,41^. For propionate, this sink comes via the reduction of pyruvate into propionate^42^. Therefore, inhibiting methane formation will lead to a shift in VFA profile, primarily in the direction of more propionate and butyrate^36,37^. In agreement with the current study, feeding lactating dairy cows a chemical inhibitor, 3-nitroxypropanol, increased rumen butyrate proportion 21% when CH_4_ emissions were reduced^36^. Additionally, valerate has been identified as reductant for archaea and can also function as a H^+^ sink^43^. The results from the current study, particularly regarding AT, are compatible with this shunting of H^+^ to other metabolic pathways when formation of CH_4_ was decreased in vitro^20,24^.

### Ammonia

Mean ammonia concentration from the present study across all treatments and sets was 7.70 mM (SD ± 2.32), ranging from 1.57 to 14.56 mM (Table 1). Compared with control, ammonia concentration was increased (*P* < 0.03) 25% by *Vertebrata lanosa*. *Sargassum horneri*, *Chondracanthus exasperatus*, and *Sargassum filipendula* all decreased (*P* ≤ 0.04) ammonia concentrations by 16, 19, and 21%, respectively. Aside from *Sargassum horneri*, these 3 species were analyzed within the same Set (13) where all algae numerically decreased ammonia concentrations, potentially indicating a higher control in Set 13 (9.07 vs. 7.90 mM; Set 13 control and all Sets control, respectively). Obvious commonality in taxonomy is shared between the 2 *Sargassum* spp., whereas *V. lanosa* and *C. exasperatus* are all classified under *Rhodophyta*.

**Table 1:** Descriptive statistics of fermentation variables across incubation sets.

Variable	N	Mean	SD	Minimum	Maximum	CV	Lower 95% CL for mean	Upper 95% CL for mean
Total gas production, mL/g of DM^1^	549	122	12.3	80.6	160.9	10.0	121	124
CH_4_ yield, mL/g of DM	505	6.73	2.75	0.05	12.85	40.9	6.49	6.97
Total VFA, µM	555	54.1	10.19	25.3	89.5	18.8	53.3	55.0
VFA, % molar proportion								
Acetate	555	58.3	4.30	42.2	67.8	7.4	58.0	58.6
Propionate	555	26.1	3.64	17.1	37.0	14.0	25.8	26.4
Isobutyrate	555	1.02	0.411	0.51	9.12	40.4	0.99	1.05
Butyrate	555	10.8	1.52	7.12	16.0	14.0	10.7	11.0
Isovalerate	55	3.48	1.133	0.73	7.91	32.5	3.39	3.57
Valerate	555	2.00	0.492	0.70	8.84	24.7	1.95	2.04
Acetate:Propionate	555	2.29	0.443	1.20	3.97	19.3	2.25	2.33
Ammonia, m*M*	550	7.70	2.316	1.57	14.54	30.1	7.50	7.89
Incubation end pH	574	6.52	0.066	6.34	6.78	1.0	6.52	6.53

^1^DM = fermentation substrate dry matter (1.5 g/vessel).

Increases in ammonia concentration, as in the case of *V. lanosa*, may simply reflect an increase in crude protein concentration in the seaweed species^31^. Chemical composition data, however, were not available to confirm this hypothesis. Decreases in ammonia concentration is an indication of enhanced ammonia-N utilization by rumen microbes, which may be limited by carbohydrate availability, or decreased proteolysis^39,44^. Given the low inclusion level of the macroalgae as substrate, it is unlikely that treatment would affect changes in fermentation (and fermentation end-products) through nutrient availability. In a similar in vitro protocol, alterations of starch source (barley vs. corn) by as much as 75% failed to illicit a significant effect on ammonia concentration in vitro^45^.

Other possible explanations for decreased ammonia concentration would be interactions with bioactive compounds found in the macroalgae that may inhibit substrate proteolysis^16,46^. For example, phlorotannins from *Ascophyllum nodosum* produced a linear decrease in ammonia concentration with increasing dose in vitro^39^. While in the current study, *A. nodosum* only numerically decreased ammonia concentration, the principle that bioactive compounds in macroalgae can affect ammonia concentration in vitro is still valid. However, it should be emphasized that in a previous study, phlorotannins were administered as an extract at minimum of 125 µg/mL; almost tenfold the dose used in the current study, assuming similar whole plant concentration of phlorotannin in *A. nodosum*^39^. Phlorotannin concentrations within macroalgae were not quantified in the current study.

### Microbial profile

In archaeal communities, a total of 157,254 raw reads were generated from 2 incubations. Quality filtering produced a total of 140,395 reads and approximately 11% of sequences were filtered. This produced 4,340 operational taxonomic units (OTU). In bacterial communities, a total of 629,162 raw reads were generated from 2 incubations. Quality filtering produced a total of 410,198 reads and approximately 35% of sequences were filtered. This produced 25,656 amplicon sequences variants.

As the main interest of this study was inhibition of methanogenesis, only AT, chloroform (CHCl_3_; positive control) and control treatments were pooled, sequenced, and analyzed for differences in total abundance (DNA) amongst bacterial and archaeal OTU. Three archaeal genera were present at greater than 1% abundance: *Methanobacteriaceae*, *Methanobrevibacter* and *Methanosphaera* (Table 2). *Methanobrevibacter* was by far the most abundant with a relative abundance between 95.0 and 96.5%. *Asparagopsis taxiformis* increased (*P* < 0.007) the prevalence of *Methanobrevibacter* from 95 to 96% abundance, while decreasing (*P* < 0.001) *Methanobacteriaceae* from 1.4 to < 0.1% abundance, compared with control. *Methanobacteriaceae* was also decreased (*P* < 0.003) by AT compared with CHCl_3_ (0.53% abundance). Compared with AT and control, CHCl_3_ decreased (*P* < 0.01) *Methanosphera* abundance by 27 and 21%, respectively.

**Table 2:** Archaeal relative abundance in incubation medium in vitro.

Archaea genus (*Euryarchaeota*)	Treatment			SEM^1^	*P* value^2^
	CON	+ CON	AT		
*Methanobacteriaceae*	1.37^a^	0.53^b^	0.07^c^	0.066	< 0.001
*Methanobacterium*	0.23	0.26	0.22	0.017	0.19
*Methanobrevibacter*	95.0^a^	96.5^b^	96.0^b^	0.19	0.004
*Methanosphaera*	3.43^a^	2.71^b^	3.66^a^	0.146	0.009

Presented as percentage of the total sequences analyzed within the sample.

CON = Control (no seaweed), + CON = Chloroform (10 µmol), AT = *Asparagopsis taxiformis* (included at 2% of feed DM).

^a,b,c^Within a row, means without a common superscript letter differ (*P* < 0.05).

^1^n = 9.

^2^Main effect of treatment.

Number of observed archaeal species was different among control, CHCl_3_, and AT pools (*P* = 0.004). Lower archaeal species richness was observed within AT samples compared with control and CHCl_3_ (Supplementary Figs. 1 and 2). Kruskal–Wallis test showed differences (*P* = 0.005) among pools, however there were no pairwise differences based on Wilcoxon test. Beta diversity PERMANOVA results differed by pool in Weighted UniFrac (*P* = 0.001); however, pairwise PERMANOVA did not differ.

Analysis of archaeal complementary DNA (cDNA) by RT-qPCR for log copy numbers of gene specific primers *Methanosphera stadtmane* [mtaB(843)]*, Methanobrevibacter ruminantium* [mcrH(835)], *Methanobrevibacter smithii* [mtaB(443)], and 16*S* rRNA revealed several differences between treatments (Supplementary Table 3). Complimentary DNA is a synthetic DNA transcribed from mRNA and can indicate the level of expression for a specific mRNA sequence within a sample [ex. mtaB(843)]^47^. Gene copy numbers of *Methanosphera* cDNA based component mtaB(843) was decreased (*P* ≤ 0.001) by AT and CHCl_3_ when compared with control (79 and 55%, respectively). Additionally, compared with control mcrH(835) was decreased (*P* < 0.001) 10% by AT, but was increased (*P* = 0.002) 5% by CHCl_3_. No differences were detected in component mtaB(443). Total 16S rRNA was decreased (*P* = 0.05) 2% by CHCl_3_. Differences between the methane inhibiting treatments were also observed; as both mtaB(843) and mcrH(835) gene copy numbers were decreased (*P* ≤ 0.05) by AT compared with CHCl_3_ (53 and 14%, respectively).

Complementary DNA analysis indicate that although AT increased DNA copy number of *Methanobrevibacter*, activity level of *M. ruminantium* was decreased, while *M. smithii* remained unaffected. In mice models utilizing human strains of *M. smithii*, non-methanogenic removal of fermentation end products (i.e. CO_2_) was observed in the form of an incomplete reductive tricarboxylic acid cycle^48^. The metabolic flexibility of *M. smithii* in this instance may explain why cDNA expression was unaffected and DNA abundance of the genus was able to increase despite inhibition of methanogenesis. Interestingly, *Methanospera* DNA abundance was not reduced by AT, but gene activity was severely reduced. A likely explanation for the response difference between DNA and cDNA could be the lack of medium passage that is specific to batch in vitro culture systems. This suggests that, as AT inhibits methanogenesis, accumulation of cDNA is reduced, but the archaeal DNA present at the time of inoculation is retained throughout the incubation. Decreases in gene activity by AT compared with CHCl_3_ may indicate that while both, CHBr_3_ and CHCl_3_ are methane analogs capable of inhibiting methanogenesis, other volatile organic compounds (e.g. dibromochloromethane) within AT are providing additive inhibition of methanogenesis^22^.

Number of observed bacterial species and Shannon diversity were not different among treatment pools, however Weighted (*P* = 0.008) and Unweighted (*P* = 0.02) beta diversity was observed between pools (Supplementary Figs. 3 and 4). No pairwise beta diversity was observed. Bacterial abundance was distributed across 12 phyla with Bacteroidetes and Firmicutes comprising 61.9 and 33.1% of the DNA in control, respectively (Table 3). Additionally, Fibrobacteres accounted for 1.4% of bacteria abundance in control. *Asparagopsis taxiformis* decreased (*P* < 0.03) the relative abundance of Bacteroidetes lineages from 61.9 to 57.6% and decreased (*P* < 0.01) Firmicutes abundance from 33.1 to 30.9%. Fibrobacteres was increased (*P* < 0.002) by AT from 1.4 to 5.6% compared with control. Spirochaetes was also increased (*P* < 0.001) by AT from 0.6 to 3.2% compared with control. Similarly, AT decreased (*P* < 0.006) Bacteroidetes, while increasing (*P* < 0.001) Fibrobacteres and Spirochaetes compared with CHCl_3_.

**Table 3:** Bacterial relative abundance by phylum in incubation medium in vitro.

Phylum	Treatment			SEM^1^	*P* value^2^
	CON	+ CON	AT		
Unassigned	0.12	0.09	0.04	0.024	0.12
Actinobacteria	0.39^ab^	0.33^b^	0.46^a^	0.026	0.03
Bacteroidetes	61.9^a^	63.9^a^	57.6^b^	1.06	0.02
Cyanobacteria	0.24^a^	0.21^ab^	0.13^b^	0.029	0.08
Fibrobacteres	1.38^b^	1.18^b^	5.56^a^	0.556	0.002
Firmicutes	33.1^a^	31.9^ab^	30.9^b^	0.44	0.04
Proteobacteria	1.26	1.09	1.27	0.116	0.50
Spirochaetes	0.58^b^	0.42^b^	3.18^a^	0.230	< 0.001
Synergistetes	0.10	0.11	0.05	0.038	0.53
TM7	0.17	0.17	0.15	0.023	0.86
Tenericutes	0.56	0.47	0.37	0.085	0.35
WPS-2	0.06	0.05	0.03	0.012	0.24

Presented as the percentage of the total sequences analyzed within the sample.

CON = Control (no seaweed), +  CON = Chloroform (10 µmol), AT = *Asparagopsis taxiformis* (included at 2% of feed DM).

^a,b,c^Within a row, means without a common superscript letter differ (*P* < 0.05).

^1^n = 9.

^2^Main effect of treatment.

At the genus level, the most abundant bacteria by far were *Prevotella* which comprised up to 42% of the bacterial abundance (Table 4). *Asparagopsis taxiformis* decreased (*P* < 0.001) *Prevotella* from 40.8 to 35.7% compared with control. Unassigned genera in the family *Bacteroidales* were the second most abundant and were decreased (*P* < 0.01) by AT from 11.7 to 8.6% compared with control. For comparison, Pitta et al. (2018) reported similar relative sequence abundance of *Bacteroidales* (11%) within the ruminal liquid fraction of cows in the same herd as the present study^49^. *Asparagopsis taxiformis* increased (*P* < 0.002) *Clostridium* and *Anaerovibrio* abundance from 0.7 to 8.0% and from 1.7 to 3.4% compared with control, respectively. Comparison of AT with CHCl_3_ had similar results. Correspondingly, AT decreased (*P* < 0.02) *Bacteriodales* and *Prevotella*, while increasing (*P* ≤ 0.004) *Clostridium* and *Anerovibrio* compared with CHCl_3_.

**Table 4:** Bacterial relative abundance by family and/or genus in incubation medium in vitro.

Bacteria genus (family, genus)	Treatment			SEM^1^	*P* value^2^
	CON	+ CON	AT		
*Bacteroidales* (unclassified)	11.7^a^	11.3^a^	8.6^b^	0.60	0.02
*Prevotellaceae Prevotella*	40.8^a^	42.4^a^	35.7^b^	0.51	< 0.001
*RF16* (unclassified)	0.65^a^	0.69^a^	1.45^b^	0.087	0.001
*S24-7* (unclassified)	1.71^a^	2.25^a^	0.65^b^	0.318	0.03
*Paraprevotellaceae* (unclassified)	1.18^a^	1.19^a^	1.60^b^	0.060	0.004
*Paraprevotellaceae CF231*	1.58^a^	1.44^b^	1.21^c^	0.022	< 0.001
*Paraprevotellaceae YRC22*	1.02^c^	1.25^b^	1.65^a^	0.041	< 0.001
*Fibrobacteraceae Fibrobacter*	1.38	1.19	1.20	0.097	0.37
*Clostridiales* (unclassified)	3.43^a^	3.54^a^	4.23^b^	0.186	0.04
*Clostridiales* (unclassified)	4.00	3.63	3.53	0.215	0.33
*Clostridiaceae Clostridium*	0.66^b^	0.51^b^	8.03^a^	0.189	< 0.001
*Lachnospiraceae* (unclassified)	1.02^b^	0.98^b^	1.52^a^	0.073	0.003
*Ruminococcaceae Ruminococcus*	3.15	2.69	2.46	0.474	0.61
*Veillonellaceae* (unclassified)	1.23^ab^	1.39^a^	0.95^b^	0.119	0.10
*Veillonellaceae Anaerovibrio*	1.36^b^	1.66^b^	3.36^a^	0.268	0.004
*Veillonellaceae Selenomonas*	1.24	1.47	1.75	0.151	0.13
*Veillonellaceae Succiniclasticum*	4.26	4.62	4.68	0.278	0.54
*Mogibacteriaceae* (unclassified)	2.27^a^	1.84^b^	1.09^c^	0.069	< 0.001
*Spirochaetaceae Treponema*	0.48^b^	0.34^b^	0.85^a^	0.062	0.003

Presented as the percentage of the total sequences analyzed within the sample.

CON  = Control (no seaweed),  + CON = Chloroform (10 µmol), AT = *Asparagopsis taxiformis* (2% of DM inclusion).

^a,b,c^Within a row, means without a common superscript letter differ (*P* < 0.05).

^1^n = 9.

^2^Main effect of treatment.

### Spearman correlations

Correlation analysis between species of bacteria and in vitro response variables (gas production and composition, VFA, and ammonia) were conducted on control, CHCl_3_, and AT pooled samples to determine relationships as affected by the inhibition of methanogenesis (Supplementary Fig. 5 and Supplementary Table 4). Thirty-one significant correlations (*r* ≥|0.5| and *P* ≤ 0.05) were observed between Bacteroidetes, Firmicutes, and Spirochaetes genera. *Bacteroidales, CF231, Clostridium* and *Mogibacteriaceae* were positively correlated with CH_4_ emission. A strong, negative correlation (*r* = − 0.70, *P* = 0.04) was observed between *YRC22* and CH_4_ emission, along with a very strong positive correlation (*r* = 0.87, *P* = 0.002) with H_2_ emission. *Paraprevotellaceae, Clostridiales, Lachnospiraceae,* and *Treponema* also had a very strong positive correlation (*r* ≥ 0.85, *P* ≤ 0.004) with H_2_ emission. These 4 species were numerically, negatively correlated to CH_4_ emission. Hydrogen emission was negatively correlated (*r* ≤ − 0.71, *P* ≤ 0.03) with *Prevotella*, *CF231*, and *Selenomonas*.

Total VFA concentration was positively associated (*r* = 0.69, *P* = 0.04) with *CF231* only. Proportion of acetate was positively correlated (r ≥ 0.68, *P* ≤ 0.04) with *CF231* and *Clostridium*, with both genera being negatively correlated (*r* ≤ − 0.69, *P* ≤ 0.04) with propionate concentration. Acetate was negatively correlated (r ≤ − 0.67, *P* ≤ 0.05) with *YRC22* and *Treponema*, again, with the inverse correlation (*r* ≥ 0.69, *P* ≤ 0.04) with propionate being observed. *Firmicutes* (*Mogibacteriaceae*) was negatively correlated (r = − 0.68, *P* = 0.04) with butyrate. Finally, valerate was positively correlated (r ≥ 0.69, *P* ≤ 0.04) with *YRC22, Paraprevotellaceae, Lachnospiraceae,* and *Treponema* and negatively correlated (r ≤ − 0.68, *P* ≤ 0.04) with *CF231, Bacteriodales* and *Mogibacteriaceae*.

The positive correlation between H_2_ and *Clostridiales* observed in this study aligns with metagenomic and metatranscriptomic data which identified several hydrogenase genes within the order’s genome^50^. Bacteria possessing these genes provide the majority of the H_2_ used for hydrogenotrophic methanogenesis. Other genera that represent a large share of hydrogenase activity in ruminants include *Clostridia, Bacteroides, Butyrivibrio, Clostridium, Sarcina*, and the Christensesnellaceae R-7 group of bacteria^50,51^. It is unclear why more correlations were not observed between these bacterial groups and H_2_ yields in the current study. Relative abundance of bacterial DNA is not a reliable indicator of metabolic activity, and metatranscriptomic data for hydrogenase genes is not available.

The correlations between bacterial communities and gas composition reflect the known inverse relationship between CH_4_ and H_2_ concentrations, as all identifiable correlations for CH_4_ and H_2_ emission were directly opposed; there were no cases of unidirectional correlation of CH_4_ and H_2_ with a bacterial genus. Only *CF231* and *YRC22* had qualifiable correlations with both CH_4_ and H_2_ emissions, suggesting that their metabolic activity may be more tightly associated with methanogenesis than the other genera investigated in this study. Interestingly, these two genera were found in higher abundance in rumen microbial communities of low-yield cows compared with high-yield; however, very little is known about their function in rumen physiology^52^. Another study analyzing cDNA-based bacterial communities in rumen samples from phenotypically high- and low-methane emitting cows observed a negative correlation between YRC22 and propionate^53^. Direct comparison between those c-DNA-based correlations and DNA-based correlations within the present study cannot be made, but nonetheless suggests some connection between YRC22 and propionate. *CF231* had the most observed correlations (7) with response variables in this study. Being positively associated with CH_4_ emission, total VFA concentration and acetate proportion, *CF231* may be preferential to a rumen environment promoting methanogenesis. Conversely, all genera that were positively correlated with H_2_ had positive correlations with propionate proportion, a known H_2_ sink. Both patterns agree with H_2_ metabolism discussed in Janssen (2010)^37^. While *CF231* and *YRC22* are genera of interest in both the present study and Mu et al. (2018)^52^, the authors are unaware of any work describing the specific role and function of these bacteria within the rumen. Future studies should be conducted to detail *CF231* and *YRC22* activity within the rumen microbiome. More work in vivo needs to be conducted to understand the influence AT may exert on animal production and the rumen microbiome, but it appears that the observed changes in rumen fermentation are driven by the thermodynamics and stoichiometry surrounding H_2_ metabolism.

## Materials and methods

### Macroalgae species

Samples for the current study were collected from April 2018 through August 2019 across several coastal US geographies (Supplementary Table 1). Red, brown, and green macroalga were selected based on multiple criteria: current or potential ability to be widely cultivated; opportunities to be cultivated within integrated multi-trophic aquaculture or nutrient bioextraction systems; potential bioactive compound content; opportunities for sustainable wild harvest; and industry interest. Fresh samples were identified, cleaned of epiphytes, rinsed with sterilized seawater and then frozen at − 20 °C for shipment. Upon delivery, samples were lyophilized (HarvestRight, North Salt Lake, Utah), ground in a Wiley mill (1-mm screen, Thomas Scientific, Swedesboro, NJ), and stored in airtight, brown glass vials at 4 °C. Before the in vitro test, samples were ball-ground using a Retsch MM200 mixer mill (Retsch, Inc., Newtown, PA).

### Donor cows

Animal used in this study were cared for according to the guidelines of the Pennsylvania State University Institutional Animal Care and Use Committee. The Pennsylvania State University Institutional Animal Care and Use Committee reviewed and approved all procedures involving animals. Animal care and reporting of data within this manuscript adheres to ARRIVE guidelines.

Pairs of 2 ruminally-cannulated (11.0 cm i.d. silicone cannulas; Robyn Williams, Victoria, Australia) Holstein cows were used as rumen inoculum donors. Cows were housed at The Pennsylvania State University’s Dairy Teaching and Research Center tie-stall barn. Due to the large number of incubations conducted over the course of 18 months, a total of 7 cows were used as donors of ruminal fluid in the study. Average lactation number, days in milk, DMI, and milk production of the cows were (mean ± SD): 3.3 ± 0.83 lactations, 238 ± 119 d, 30.5 ± 4.86 kg/d, and 48.6 ± 8.38 kg/d, respectively. Cows had free access to drinking water and diets were fed from a Rissler model 1050 TMR mixer (I.H. Rissler Mfg. LLC, Mohnton, PA). Feeding was once daily at around 0900 h, after the morning milking, and feed was offered ad libitum targeting 10% refusals. Animals were fed a typical TMR containing (%, DM): corn silage (39), alfalfa haylage (12), canola meal (11), ground corn grain (10), roasted whole soybeans (8), cookie meal (7), whole cottonseed (5), sugar (5), grass hay (2), and mineral/Optigen^®^ (Alltech, Nicholasville, KY) mix (2). A composite TMR sample was oven dried at 55 °C for 72 h and submitted to Cumberland Valley Analytical Services Inc. for wet chemistry analyses of CP^54^, α-NDF^55^, ADF^54^, starch^56^, minerals^54^, and estimated net energy for lactation (NE_L_)^57^. Nutrient composition of the TMR (%, DM basis) was: crude protein, 16.5; NDF, 30.9; ADF, 21.0; starch, 25.5; NE_L_, 1.61 Mcal/kg DM: Ca, 0.81; and P, 0.43.

### Preparation of rumen inoculum

Collections of whole rumen contents took place before feeding and the morning milking at approximately 0500 h. Contents were collected from the ventral sac, reticulum, and caudal and dorsal sections of the feed mat and processed as previously described^43^. Briefly, contents were filtered through 2 layers of cheesecloth, reserving the filtrate. Strained solids were then combined with McDougal’s buffer^58^ and vigorously shaken for 30 s to extract loosely-associated microbes. The buffer and filtrate were then combined in equal parts and placed into a prewarmed thermos. Inoculum was transported back to the lab within 20 min of collection. Once at the laboratory, inoculum was transferred into 2 L graduated cylinders and permitted to ferment for 45 min under CO_2_ at 39 °C; buoyant feed particles were removed by vacuum aspiration and the remaining ruminal inoculum was used for the incubation. Aspiration of these particles improves the uniformity of the inoculum and prevents the addition of unaccounted substrate to the incubation vessels.

### In vitro incubation

**Figure 5:**
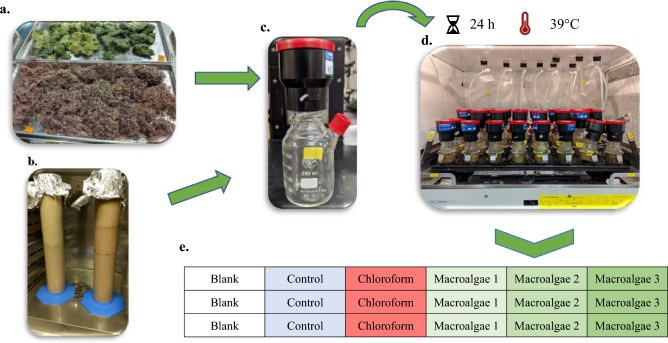
Incubation workflow overview. Incubation workflow: (1) Macroalgae (**a**) and feed substrate are dried, ground, and added to the vessels (**c**) with the buffer; (2) Rumen inoculum (**b**) is collected and clarified; (3) Clarified inoculum is added to the vessels; (4) Vessels are sealed and placed into the incubator (**d**). Incubation layout example (**e**): Blank (no substrate), Control (feed substrate only), Chloroform (feed substrate + CHCl_3_), and Macroalgae (feed substrate with 2% DM Macroalgae).

Incubations were conducted with the Ankom RF Gas Production System (Ankom Technology, Macedon, NY) outfitted with 250 mL glass vessels. Incubations were carried out for 24-h in a New Brunswick Innova 44 incubator/shaker (Eppendorf North America, Enfield, CT) at 39 °C and continuous 75 rpm agitation. A total of 30 incubations were completed. Each incubation was replicated and together is referred to as a “Set”. All treatments were run in triplicate within an incubation and all sets contained; TMR only (control), TMR + CHCl_3_ (positive control), and treatments (TMR + macroalgae) vessels. Prior to inoculation, TMR and, where appropriate, inhibitor or macroalgae (totaling 1.5 g DM) were weighed into the vessels and soaked in 75 mL of McDougall’s buffer for 1.5 to 2.0 h at 39 °C (Fig. 5). Upon conclusion of inoculum processing, each incubation vessel received 75 mL of ruminal inoculum dispensed from a continuously stirred flask, purged with CO_2_, and kept on a warming plate set at 39 °C. For positive control treatments, 0.5 mL of CHCl_3_ and buffer solution (0.81 µL/mL) was added. The CHCl_3_ (99% pure; Sigma Aldrich, St. Louis, MO) was dissolved in McDougall’s buffer and added to the TMR and buffer mixture just before the addition of rumen inoculum. Chloroform concentration was chosen to deliver a CH_4_ inhibition of around 80%^25^. Total inoculum volume of all vessels was 150 mL.

A 2% inclusion level was selected for the algal treatments since AT has been shown to be effective at decreasing CH_4_ emission at 2% of DM in vitro^20–23^. The first 11 sets contained 3 species/set at multiple inclusion levels (< 2% DM basis); but these lower inclusion rates did not produce any measurable effect on CH_4_ emission and were excluded from the final analysis. Only the 2% inclusion level will be discussed herein. Subsequent sets (12 to 15) only tested algal biomass at 2% inclusion, allowing for 7 species to be screened per set. Initially (incubation sets 1 to 2), AT which was used in an ongoing in vivo study was used as a positive control. Due to a significant decrease in the antimethanogenic activity of AT^9^, CHCl_3_ was substituted as a positive control at 10 µmol for incubation sets 3 to 15. All data discussing AT within this manuscript refer to a separate sample of AT (containing 2.44 mg CHBr_3_/g DM) tested in set 6 that was collected with the rest of the algal samples (Supplementary Table 1).

### Sample collection and analysis

Once vessels were inoculated, initial pH was measured (Accumet AR15 pH meter; Fisher Scientific, Waltham, MA), vessels were purged with CO_2_, sealed, and placed back in the incubator. Vessels were incubated for 24-h and removed from the incubator for headspace gas sampling at 12 and 24-h according to ANKOM^RF^ Gas Production System Operator’s Manual, Appendix C- Head Space Analysis. Vessels were removed from the incubator in batches of 12 to minimize temperature flux during sampling. Gas samples (2 aliquots of 2 mL each) were removed from each vessel and placed into vacuumed 20 mL vials (Agilent Headspace screw-top, Agilent Technologies, Santa Clara, CA). Vials were then pressurized with 22 mL of ultra-high purity (UHP) N_2_ (999.99 g/kg N_2_; Praxair Inc., Danbury, CT) for a total volume of 24 mL and were either immediately analyzed for CH_4_ and H_2_ or stored at 2 °C and analyzed within 48 h using gas chromatography (GC; Agilent 7980B, Agilent Technologies). Vials were agitated for 1 min (250 rpm) at 40 °C before injection by a PAL RSI 85 autosampler (CTC Analytics, Zwingen, Switzerland) into the GC. For CH_4_ analysis, samples were injected into a HayeSep Q 80–100 mesh column (1.83 m × 2 mm; Agilent Technologies) at 310 kPa using UHP He (999.99 g/kg He; Praxair Inc.) as a carrier. A deactivated fused silica restrictor (3 m × 320 µm) operating at 58.6 kPa was used leading to flame ionization detector set at 300 °C. Hydrogen analysis was conducted using UHP N_2_ (999.99 g/kg N_2_; Praxair Inc.) as a carrier using a HayeSep Q 80–100 mesh column at a flow rate of 5 to 12 mL/min pre- and post-run, respectively. Results were obtained using a thermal conductivity detector at a flow rate of 5 mL/min at 250 °C. Handmade gas standards (35 to 7000 mg/m^3^ for CH_4_ and 4 to 900 mg/m^3^ for H_2_) were used. Standards were made via serial dilution with UHP N_2_, using chemically pure CH_4_ (99.0% purity; Praxair Inc.) and 4.5 grade H_2_ (99.995% purity; Praxair Inc.). Methane and H_2_ production were then calculated by multiplying cumulative gas production by the concentrations of the individually analyzed gases.

At incubation endpoint (24-h), vessels were sampled for gas composition as described above. The module assembly recorded temperature and cumulative pressure throughout the experiment. Immediately following gas sampling, final pH was measured for each vessel, which were then placed in an ice bath to cease fermentation and for additional sampling. Liquid aliquots were collected and analyzed for ammonia concentration by colormetric assay^59^. Liquid samples for VFA analysis were collected according to Yang and Varga (1989)^60^ and analyzed by gas chromatography (Agilent 7890B; Agilent Technologies) using an 80/100 Chromosorb WAW packed column with ultra-high purity N_2_ (999.99 g/kg N_2_; Praxair Inc.) as a carrier^61^. Results were obtained using a flame ionization detector set at 175 °C. Additional 5 mL liquid samples were collected for bacterial and archaeal abundance analysis and stored frozen at -80 °C.

### DNA extraction, 16S rRNA sequencing and bioinformatics

Replicates of control, CHCl_3_, and AT treatments from Set 6 (6 replicated per treatment) were pooled and genomic DNA was extracted in triplicate from 250 mL of each pooled sample using the repeated bead beating and column (RBB + C) method followed by extraction with a commercial kit (QIAmp Fast DNA Stool Mini Kit; Qiagen Sciences, Germantown, MD) as described in Yu and Morrison (2004)^62^. *Asparagopsis taxiformis* was the only macroalgae treatment that was extracted for DNA and 16S rRNA sequencing, as it was the most effective at reducing methane emission. The details related to library preparation and bioinformatics methodology are described in and Kaplan-Shabtai et al. (2021)^63^. Briefly, all samples were extracted for total genomic DNA, PCR-amplified for the V1-V2 region of the 16S rRNA bacterial gene, and the V6-V8 region of the archaeal 16S rRNA gene with Illumina MiSeqplatform (San Diego, CA). The sequencing reads analyzed for bacterial diversity using the QIIME2 pipeline and archaeal diversity QIIME 1.8.0 pipeline followed by statistical analysis in R (https://www.R-project.org/)^64,65^. The default parameters were used for all tools used during the analyses unless otherwise specified. For archaeal diversity analysis, the paired-end Illumina reads were joined together using combine_barcodes.py script with a 35 base-pairs overlap. The merged sequences were demultiplexed and quality filtered. Reads were discarded if they did not match the expected sample-specific barcode and 16S primer sequences (forward and reverse primers), or if they contained two or more ambiguous base calls. Reads were also discarded below a Phred quality score of 19.

### RT-qPCR analysis

Absolute quantification of ruminal methanogens based on the gene copy numbers was performed by RT-qPCR. Quantification was conducted for genomic DNA samples using a StepOnePlus Real-Time PCR System (Applied Biosystems) with Maxima SYBR Green qPCR Master Mix (2 × ; Thermo Fisher Scientific). The primers for methanogen specific genes and reference gene (16S rRNA) used in this study are listed and summarized in Pitta et al. (2021)^66^ and the methodology for RT-qPCR conditions was followed as described by Bayer et al., (2014)^67^. Briefly, the qPCR cycling steps consisted of initial DNA denaturation at 95 °C for 10 min, followed by final denaturation at 95 °C for 15 s, primer annealing at 56 °C for 30 s, and finally PCR extension at 72 °C for 30 s were set up to 40 repeated cycles, along with a melting curve to ensure the specific product amplification. Simultaneously, a tenfold dilution of full-length amplicons of the 16S rRNA reference gene was performed to generate a standard curve. All qPCR assays were performed in triplicate, and a negative (non-template) control was also set with each assay to detect non-specific fluorescence emission^68^. Analysis of raw data obtained from the assays, qPCR efficiency and gene copy numbers were done using Applied Biosystems StepOne Real-Time PCR Software v. 2.0. The amplification efficiency (98–99%) of the qPCR standard curve (16S copy number) was used to calculate the copy numbers of unknown samples.

### Sequence data processing and statistical analysis

The archaeal 16S rRNA reads were analyzed using the QIIME 1.8.0 pipeline^65^ as described in Pitta et al. (2021)^66^. The paired-end Illumina reads were joined together using combine_barcodes.py script. The merged sequences were demultiplexed and quality filtered. The operational taxonomic units (OTUs were formed by clustering sequences based on a 97% similarity threshold using the UCLUST algorithm^69^. Singleton OTU were excluded, and representative sequences for each OTU were aligned with PyNast^70^. The resultant multiple sequence alignment was used to infer a phylogenetic tree with FastTree^71^. Taxonomic assignments within the GreenGenes taxonomy^72^ were generated using the RDP Classifier version 2.2^73^. Alpha diversity was assessed via observed species and Shannon diversity and beta diversity was measured using weighted and unweighted UniFrac distances for both archaeal and bacterial communities.

Data were analyzed within set as a completely randomized design using the MIXED procedure of SAS (version 9.4; SAS Institute, Inc., Cary NC). The model included treatment as a fixed effect. Incubation was considered a random effect. Excluding microbial abundance data, treatment response variables were expressed as the percent difference from control replicate averages within set; the averaged differences were used for the statistical analysis. The measured alpha diversity matrices were compared between the treatment groups using the Wilcoxon/Kruskal–Wallis Rank Sum Test. For beta diversity matrices, a non-parametric permutational multivariate ANOVA test^74^, implemented in the vegan package for R, was used to test the interactions and main effects. Microbial relative abundance percentages were compared directly, as samples were pooled by treatment (i.e., control, CHCl_3_, AT) before analysis. Spearman correlation between bacterial genera with a sequence proportion of ≥ 0.01% relative abundance and fermentation parameters were conducted on pooled control, CHCl_3_, and AT treatments using R. Statistical differences were considered significant at *P* ≤ 0.05 and trends were declared at 0.05 < *P* ≤ 0.10. Gas and fermentation response data are presented as LSM. Correlations were declared at *P* ≤ 0.05 with *r* values above 0.50 and below -0.50.

## Conclusions

Of the 67 species of macroalgae investigated in this in vitro study, *A. taxiformis* was the only species that had a substantial mitigating effect on CH_4_ emission. Although *Colpomenia peregrina* also decreased CH_4_ emissions, this relatively smaller reduction (< 20%) has yet to be replicated in vivo. The reduction in CH_4_ by *A. taxiformis* was accompanied by a decrease in total gas production and total VFA concentration, indicating, at the 2% inclusion rate, a negative effect on overall fermentation. Diminishing the role of CH_4_ as a H_2_ sink resulted in a decrease in molar proportion of acetate, and an increase in molar proportions of propionate, butyrate, and valerate. No effect of *A. taxiformis* on ammonia concentration was observed; however, several other macroalgae decreased ammonia concentrations 16 to 21%. Additionally, *Vertebrata lanosa* increased ammonia concentration 25%. *Asparagopsis taxiformis* increased archaeal abundance of *Methanobrevibacter* while decreasing *Methanobacteriaceae,* and decreased bacterial abundance of *Prevotella* while increasing *Clostridium*. In the conditions of this in vitro study, no other macroalgae approached the efficacy of *A. taxiformis* in terms of CH_4_ emission mitigation. By altering the utilization of H_2_, *A. taxiformis* influenced the profile and gene expression level of bacterial and archaeal communities, shifting their metabolism to favor the production of VFAs which act as H_2_ sinks. Two specific bacterial species, CF231 and YRC22, were highly correlated with CH_4_ and H_2_ emission when methanogenesis was inhibited by *A. taxiformis*. Further investigation into the metabolic role of CF231 and YRC22 within an inhibited rumen environment is warranted. No other macroalgae, except for *A. taxiformis*, demonstrated the potential to be used as a CH_4_ mitigation tool for livestock in this study.

## Supplementary Information

Supplementary Information.

## Data Availability

The raw bacterial 16 s rRNA sequencing data are available at NCBI (National Center for Biotechnology Information) BioProject with accession number PRJNA950482.
