# Supplementary material for: Screening macroalgae for mitigation of enteric methane in vitro
Source: Sci Rep. 2023 Jun 17;13:9835. doi: 10.1038/s41598-023-36359-y (PMC10276865; doi:10.1038/s41598-023-36359-y)
Supplement: Supplementary file 1 — Supplementary Information. [file 41598_2023_36359_MOESM1_ESM.docx]

**
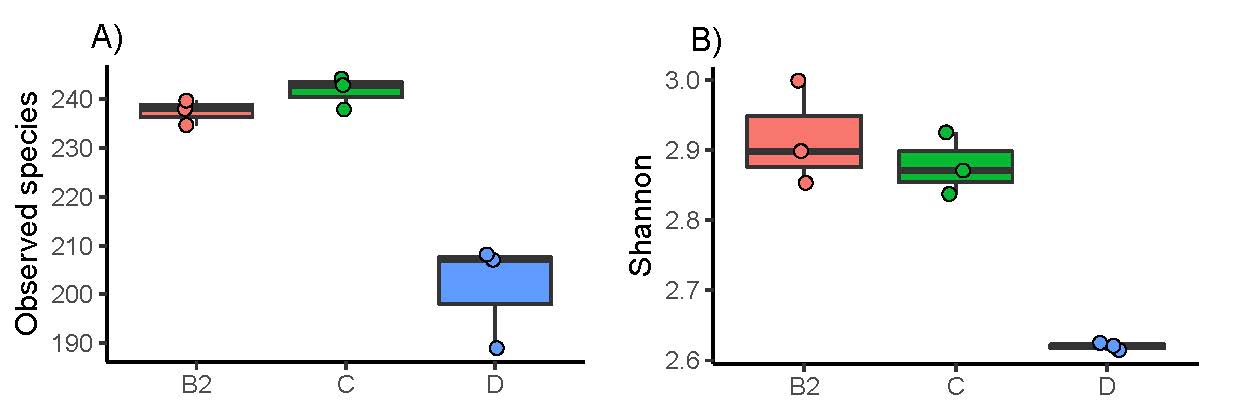
**

**Supplementary Figure 1.** Comparison of archaeal α diversity measures (A, observed species and B, Shannon diversity) between treatment groups (B2 = Control, C = Chloroform, and D = *Asparagopsis* *taxiformis* treatment) for DNA. Boxes represent the interquartile range (IQR) between first and third quartiles, and the horizontal line inside the box defines the median. Whiskers represent the lowest and highest values within 1.5 times the IQR from the first and third quartiles, respectively.

**
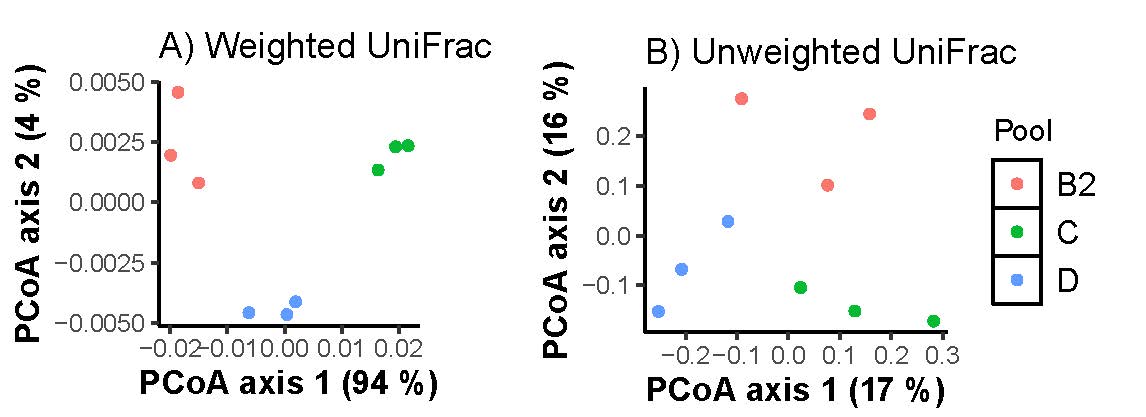
**

**Supplementary Figure 2**. Comparison of archaeal community composition between treatment groups (B2 = Control, C = Chloroform, D = *Asparagopsis taxiformis*) using principal coordinate analysis (PCoA). (a) Weighted UniFrac distances based on relative abundance of archaeal operational taxonomic units (OTU) and (b) unweighted UniFrac distances based on presence or absence information or archaeal OTU.


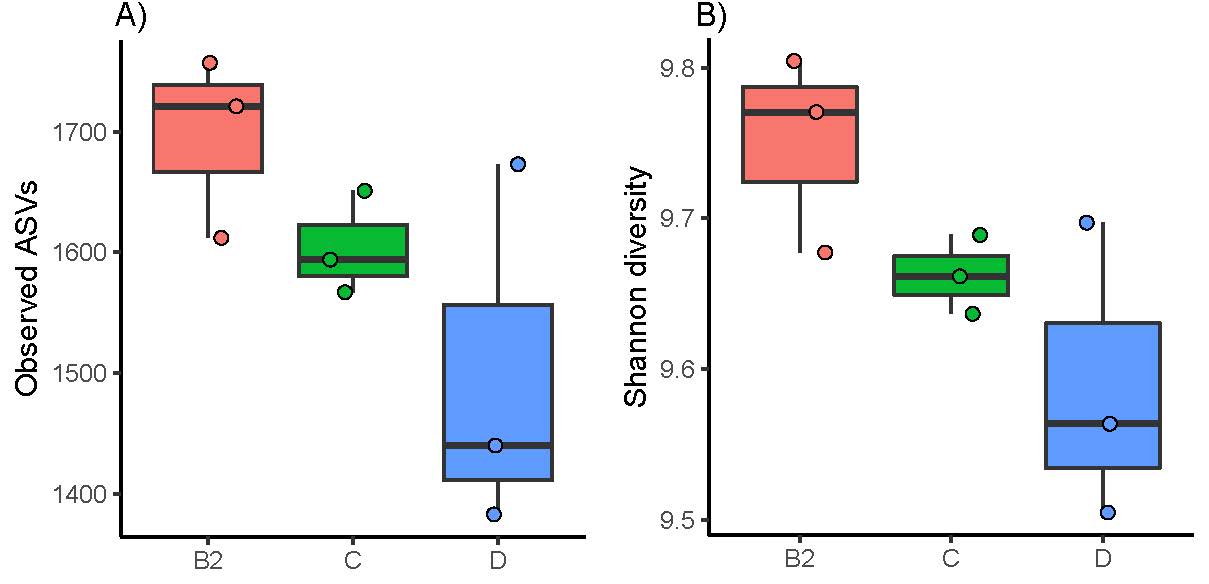


**Supplementary Figure 3.** Comparison of bacterial α diversity measures (A, observed species and B, Shannon diversity) between treatment groups (B2 = Control, C = Chloroform, and D = *Asparagopsis taxiformis*) for DNA. Boxes represent the interquartile range (IQR) between first and third quartiles, and the horizontal line inside the box defines the median. Whiskers represent the lowest and highest values within 1.5 times the IQR from the first and third quartiles, respectively.


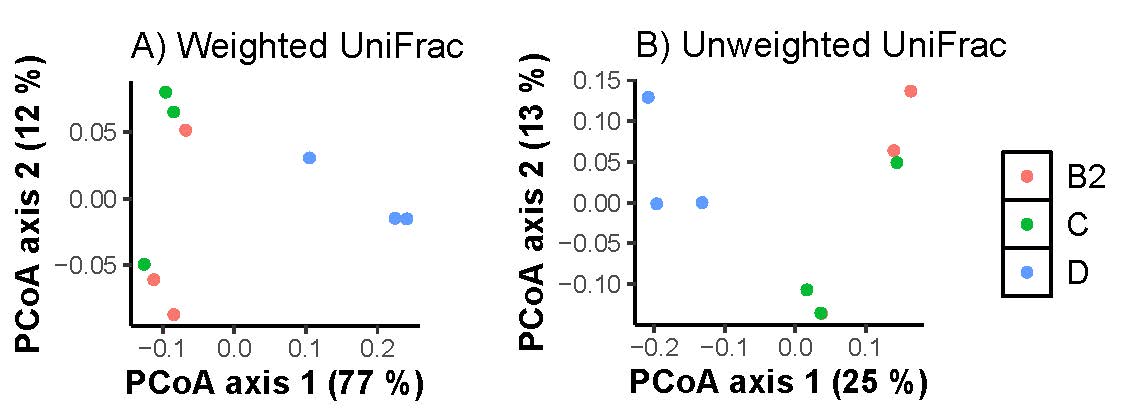


**Supplementary Figure 4.** Comparison of bacterial community composition between treatment groups (B2 = Control, C = + Control, D = *Asparagopsis taxiformis*) using principal coordinate analysis (PCoA). (a) Weighted UniFrac distances based on relative abundance of archeal operational taxonomic units (OTU) and (b) unweighted UniFrac distances based on presence or absence information or archaeal OTU.


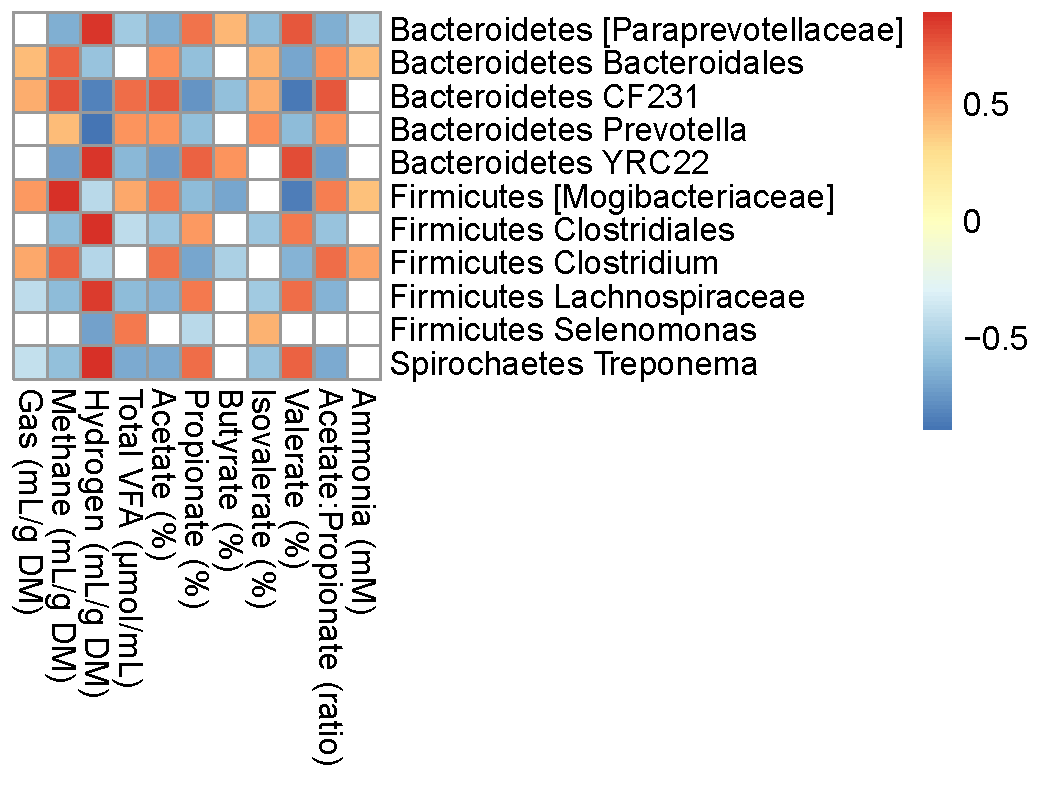


**Supplementary Figure 5.** Analysis of association patterns among microbial lineages and fermentation parameters scored using Spearman correlation. Correlations are shown by the color code (red: positive correlations and blue: negative correlations.

Supplementary Table 1. Macroalgae set order and collection locations

| Set | ID | Macroalgae | Collection location |
| --- | --- | --- | --- |
| 1 | 1 | *Ascophyllum nodosum* | Avery Point, Groton, CT |
|  | 2 | *Ecklonia arborea* | Santa Catalina Island, CA |
|  | 3 | *Sargassum horneri* | Santa Catalina Island, CA |
| 2 | 1 | *Fucus vesiculosus* | Avery Point, Groton, CT |
|  | 2 | *Mastocarpus papillatus* | Bodega Bay, CA |
|  | 3 | *Sargassum fluitans* | Cancun, Mexico |
| 3 | 1 | *Chondrus crispus* | Avery Point, Groton, CT |
|  | 2 | *Dictyopteris undulata* | Santa Catalina Island, CA |
|  | 3 | *Pelvetiopsis limitata* | Bodega Bay, CA |
| 4 | 1 | *Macrocystis pyrifera* | Santa Catalina Island, CA |
|  | 2 | *Neoporphyra perforata* | Bodega Bay, CA |
|  | 3 | *Solieria tenera* | Santa Catalina Island, CA |
| 5 | 1 | *Egregia menziesii* | Bodega Bay, CA |
|  | 2 | *Neorhodomela larix* | Bodega Bay, CA |
|  | 3 | *Odonthalia floccosa* | Bodega Bay, CA |
| 6 | 1 | *Asparagopsis taxiformis* | Santa Catalina Island, CA |
|  | 2 | *Endocladia muricata* | Bodega Bay, CA |
|  | 3 | *Prionitis lanceolata* | Bodega Bay, CA |
| 7 | 1 | *Corallina officinalis* | Swan’s Island, ME |
|  | 2 | *Fucus spiralis* | Swan’s Island, ME |
|  | 3 | *Laminaria farlowii* | Goleta, CA |
| 8 | 1 | *Colpomenia peregrina* | Swan’s Island, ME |
|  | 2 | *Devaleraea ramentacea* | Swan’s Island, ME |
|  | 3 | *Fucus evanescens* | Swan’s Island, ME |
| 9 | 1 | *Ectocarpus siliculosus* | Swan’s Island, ME |
|  | 2 | *Spermothamnion repens* | Swan’s Island, ME |
|  | 3 | *Ulva intestinalis* | Bodega Bay, CA |
| 10 | 1 | *Ceramium rubrum* | Swan’s Island, ME |
|  | 2 | *Spongomorpha aeruginosa* | Swan’s Island, ME |
|  | 3 | *Ulva sp.* | Swan’s Island, ME |
| 11 | 1 | *Colpomenia sp.* | Swan’s Island, ME |
|  | 2 | *Polysiphonia sp.* | Swan’s Island, ME |
|  | 3 | *Vertebrata lanosa* | Swan’s Island, ME |

Supplementary Table 1 (cont.). Macroalgae set order and collection locations

| Set | ID | Macroalgae | Collection location |
| --- | --- | --- | --- |
| 12 | 1 | *Chaetomorpha linum* | Avery Point, Groton, CT |
|  | 2 | *Chorda filum* | Nauset Light Beach, Eastham, MA |
|  | 3 | *Cladophora sericea* | Avery Point, Groton, CT |
|  | 4 | *Colpomenia peregrina* | Swan’s Island, ME |
|  | 5 | *Eucheuma isoforme* | Santa Catalina Island, CA |
|  | 6 | *Laminaria farlowii* | Goleta, CA |
|  | 7 | *Laurencia sp.* | Santa Catalina Island, CA |
|  | 8 | *Plocamium violaceum* | Bodega Bay, CA |
|  | 9 | *Pyropia sp. (Tidal pools)* | Swan’s Island, ME |
| 13 | 1 | *Ahnfeltiopsis linearis* | Bodega Bay, CA |
|  | 2 | *Botryoglossum farlowianum* | Bodega Bay, CA |
|  | 3 | *Champia parvula* | Avery Point, Groton, CT |
|  | 4 | *Chondracanthus corymbiferus* | Bodega Bay, CA |
|  | 5 | *Chondracanthus exasperatus* | Bodega Bay, CA |
|  | 6 | *Codium fragile* | Nauset Light Beach, Eastham, MA |
|  | 7 | *Pikea californica* | Bodega Bay, CA |
|  | 8 | *Sargassum filipendula* | Avery Point, Groton, CT |
|  | 9 | *Ulva ohnoi* | Santa Catalina Island, CA |
| 14 | 1 | *Agarum clathrum* | Fort Stark, Portsmouth, New Hampshire |
|  | 2 | *Galaxaura rugosa* | Arrecife Media Luna, Lajas, Puerto Rico |
|  | 3 | *Laminaria digitata* | Newcastle, New Hampshire |
|  | 4 | *Palmaria palmata* | Newcastle, New Hampshire |
|  | 5 | *Phyllophora pseudoceranoides* | Groton, CT |
|  | 6 | *Pyropia spp. (high intertidal)* | Swan’s Island, ME |
|  | 7 | *Pyropia spp. (tidepools)* | Swan’s Island, ME |
|  | 8 | *Sarcodiotheca gaudichaudii* | Bodega Bay, CA |
|  | 9 | *Ulva (blades)* | Swan’s Island, ME |
| 15 | 1 | *Avrainvillea elliottii* | Arrecife Media Luna, Lajas, Puerto Rico |
|  | 2 | *Dictyota sp.* | Cayo San Cristobal, Lajas, Puerto Rico |
|  | 3 | *Gracilaria cervicornis* | Arrecife Media Luna, Lajas, Puerto Rico |
|  | 4 | *Halimeda monile* | Arrecife Media Luna, Lajas, Puerto Rico |
|  | 5 | *Trichogloeopsis pedicellata* | Arrecife Media Luna, Lajas, Puerto Rico |
|  | 6 | *Udotea flabellum* | Arrecife Media Luna, Lajas, Puerto Rico |
|  | 7 | *Udotea wilsonii* | Arrecife Media Luna, Lajas, Puerto Rico |

Supplementary Table 2. Effect of macroalgae on gas production and composition in vitro

| Set | ID^2^ | Item^1^ | | |
| --- | --- | --- | --- | --- |
|  |  | Total Gas Production (mL/g of DM) | CH_4_  (mmol**/**L) | CH_4_ Yield (mL/g of DM) |
| 1 | CON | 127.4 | 323.6 | 7.97 |
|  | 1 | 125.0 | 296.9 | 8.04 |
|  | 2 | 127.9 | 316.0 | 7.88 |
|  | 3 | 114.7* | 309.6 | 6.85 |
|  | SEM | 4.15 | 24.76 | 0.815 |
|  | *P* - value | 0.12 | 0.89 | 0.65 |
| 2 | CON | 125.7 | 408.6 | 9.80 |
|  | 1 | 129.0 | 388.7 | 9.60 |
|  | 2 | 130.5 | 424.5 | 10.65 |
|  | 3 | 131.4 | 419.9 | 10.60 |
|  | SEM | 2.89 | 26.68 | 0.541 |
|  | *P* - value | 0.46 | 0.77 | 0.370 |
| 3 | CON | 129.8 | 305.0 | 7.61 |
|  | +CON | 113.5* | 46.5* | 1.03* |
|  | 1 | 130.7 | 325.6 | 8.21 |
|  | 2 | 127.7 | 317.3 | 7.88 |
|  | 3 | 128.1 | 303.4 | 7.97 |
|  | SEM | 2.47 | 19.10 | 0.514 |
|  | *P* - value | < 0.001 | < 0.001 | < 0.001 |
| 4 | CON | 126.2 | 251.6 | 6.13 |
|  | +CON | 115.7* | 58.3* | 1.22* |
|  | 1 | 125.6 | 267.2 | 6.46 |
|  | 2 | 124.4 | 258.0 | 6.15 |
|  | 3 | 123.9 | 280.1 | 6.59 |
|  | SEM | 2.44 | 16.32 | 0.367 |
|  | *P* - value | 0.03 | < 0.001 | < 0.001 |
| 5 | CON | 134.1 | 343.0 | 8.90 |
|  | +CON | 128.0 | 150.1* | 3.68* |
|  | 1 | 136.9 | 362.8 | 9.31 |
|  | 2 | 131.8 | 336.9 | 8.85 |
|  | 3 | 135.3 | 345.3 | 8.96 |
|  | SEM | 5.32 | 47.26 | 1.251 |
|  | *P* - value | 0.67 | 0.00 | 0.004 |

Supplementary Table 2 (cont.). Effect of macroalgae on gas production and composition in vitro

| 6 | CON | 130.4 | 319.1 | 7.99 |
| --- | --- | --- | --- | --- |
|  | +CON | 120.0* | 112.4* | 2.66* |
|  | 1 | 112.4* | 5.1* | 0.11* |
|  | 2 | 132.3 | 304.6 | 7.76 |
|  | 3 | 130.6 | 280.7 | 7.06 |
|  | SEM | 3.15 | 26.52 | 0.608 |
|  | *P* - value | < 0.001 | < 0.001 | < 0.001 |
| 7 | CON | 118.9 | 334.8 | 7.71 |
|  | +CON | 119.4 | 16.7* | 0.39* |
|  | 1 | 123.3 | 304.8 | 7.31 |
|  | 2 | 124.7 | 292.2* | 6.99 |
|  | 3 | 117.1 | 332.5 | 7.56 |
|  | SEM | 6.20 | 25.89 | 0.856 |
|  | *P* - value | 0.84 | < 0.001 | < 0.001 |
| 8 | CON | 120.6 | 344.3 | 7.85 |
|  | +CON | 116.6 | 12.0* | 0.28* |
|  | 1 | 125.4 | 319.7 | 7.65 |
|  | 2 | 124.8 | 298.8* | 7.29 |
|  | 3 | 125.6 | 312.6 | 7.52 |
|  | SEM | 3.21 | 17.85 | 0.464 |
|  | *P* - value | 0.23 | < 0.001 | < 0.001 |
| 9 | CON | 137.4 | 319.5 | 8.48 |
|  | +CON | 131.7 | 12.7* | 0.32* |
|  | 1 | 140.4 | 295.7 | 8.04 |
|  | 2 | 134.9 | 315.9 | 8.29 |
|  | 3 | 139.5 | 318.3 | 8.65 |
|  | SEM | 3.66 | 12.13 | 0.464 |
|  | *P* - value | 0.46 | < 0.001 | < 0.001 |
| 10 | CON | 122.7 | 390.0 | 9.06 |
|  | +CON | 118.5 | 37.0* | 0.84* |
|  | 1 | 125.8 | 372.7 | 9.01 |
|  | 2 | 121.4 | 386.2 | 8.98 |
|  | 3 | 125.4 | 377.7 | 9.16 |
|  | SEM | 3.25 | 20.60 | 0.617 |
|  | *P* - value | 0.46 | < 0.001 | < 0.001 |

Supplementary Table 2 (cont.). Effect of macroalgae on gas production and composition in vitro

| 11 | CON | 127.2 | 313.3 | 7.47 |
| --- | --- | --- | --- | --- |
|  | +CON | 116.3 | 34.8* | 0.74* |
|  | 1 | 132.6 | 322.4 | 8.46 |
|  | 2 | 131.7 | 323.9 | 8.23 |
|  | 3 | 123.6 | 326.4 | 7.79 |
|  | SEM | 4.70 | 21.89 | 0.670 |
|  | *P* - value | 0.09 | < 0.001 | < 0.001 |
| 12 | CON | 122.6 | 317.4 | 7.51 |
|  | +CON | 100.9* | 27.1* | 0.53* |
|  | 1 | 123.7 | 297.6 | 7.11 |
|  | 2 | 126.7 | 269.5* | 6.57 |
|  | 3 | 129.0 | 322.3 | 8.02 |
|  | 4 | 123.5 | 272.9* | 6.51* |
|  | 5 | 121.0 | 297.3 | 6.92 |
|  | 6 | 114.5 | 297.2 | 6.54 |
|  | 7 | 120.2 | 285.6 | 6.77 |
|  | 8 | 122.9 | 320.2 | 7.57 |
|  | 9 | 121.9 | 312.5 | 7.34 |
|  | SEM | 3.13 | 20.43 | 0.560 |
|  | *P* - value | < 0.001 | < 0.001 | < 0.001 |
| 13 | CON | 120.6 | 345.8 | 8.01 |
|  | +CON | 103.3* | 55.7* | 0.966* |
|  | 1 | 127.0 | 336.4 | 8.21 |
|  | 2 | 128.2 | 326.1 | 8.07 |
|  | 3 | 124.8 | 332.3 | 7.98 |
|  | 4 | 125.8 | 333.7 | 8.09 |
|  | 5 | 128.5 | 339.5 | 8.40 |
|  | 6 | 128.3 | 339.9 | 8.37 |
|  | 7 | 132.0* | 320.5* | 8.16 |
|  | 8 | 126.7 | 329.1 | 7.98 |
|  | 9 | 125.4 | 340.9 | 8.17 |
|  | SEM | 3.53 | 14.04 | 0.446 |
|  | *P* - value | < 0.001 | < 0.001 | < 0.001 |

Supplementary Table 2 (cont.). Effect of macroalgae on gas production and composition in vitro

| 14 | CON | 122.0 | 310.1 | 7.28 |
| --- | --- | --- | --- | --- |
|  | +CON | 111.8* | 47.4* | 0.96* |
|  | 1 | 119.4 | 313.0 | 7.29 |
|  | 2 | 119.3 | 278.1* | 6.41 |
|  | 3 | 115.5 | 297.8 | 6.62 |
|  | 4 | 119.1 | 317.4 | 7.28 |
|  | 5 | 126.7 | 315.7 | 7.71 |
|  | 6 | 119.0 | 286.1 | 6.58 |
|  | 7 | 115.7 | 324.0 | 7.10 |
|  | 8 | 121.0 | 300.4 | 6.98 |
|  | 9 | 123.4 | 299.9 | 7.13 |
|  | SEM | 4.20 | 14.42 | 0.439 |
|  | *P* - value | 0.37 | < 0.001 | < 0.001 |
| 15 | CON | 97.0 | 296.3 | 5.52 |
|  | +CON | 90.9 | 81.5* | 1.40* |
|  | 1 | 103.8 | 311.9 | 5.97 |
|  | 2 | 105.0 | 245.9* | 4.99 |
|  | 3 | 102.2 | 316.9 | 6.17 |
|  | 4 | 100.3 | 289.2 | 5.57 |
|  | 5 | 105.6 | 310.1 | 6.33 |
|  | 6 | 104.1 | 307.1 | 6.15 |
|  | 7 | 103.9 | 288.5 | 5.76 |
|  | SEM | 3.01 | 18.77 | 0.395 |
|  | *P* - value | 0.01 | < 0.001 | < 0.001 |

^1^DM = Dry matter (1.5 g per vessel).

^2^CON = control (no seaweed), +CON = Chloroform; ID number corresponds to species listed in Supplementary Table 1; Largest SEM used; *P* – value = Main effect of treatment.

* Means marked with an asterisk differ from set-specific control (*P* < 0.05).

Supplementary Table 3. Archaeal gene expression

| Gene^1^ | Treatment^2^ | | | SEM^3^ | *P –* value^4^ |
| --- | --- | --- | --- | --- | --- |
|  | CON | +CON | AT |  |  |
| mtaB843 | 2.01^a^ | 0.90^b^ | 0.42^c^ | 0.141 | < 0.001 |
| mcrH835 | 8.14^b^ | 8.52^a^ | 7.31^c^ | 0.051 | < 0.001 |
| mtaB443 | 2.38 | 2.41 | 2.17 | 0.148 | 0.49 |
| 16S | 12.36^a^ | 12.07^b^ | 12.22^ab^ | 0.47 | 0.02 |

^1^Archaeal species specific gene markers; mtaB843 = *Methanosphaera stadtmane,* mcrH835 = *Methanobrevibacter ruminantium,* mtaB443 = *Methanobrevibacter smithii.*

^2^CON = Control, +CON = Chloroform, AT = *Asparagopsis taxiformis.*

^3­­^Largest SEM shown; n = 9.

^4^Main effect of treatment.

Supplementary Table 4. Correlation Coefficients between bacterial abundance and in vitro fermentation responses^1^

| Item^2^ | Bacteria (Order, Family) | | | | | | | | | | | | |
| --- | --- | --- | --- | --- | --- | --- | --- | --- | --- | --- | --- | --- | --- |
|  | *Bacteroidetes* | | | | | | *Firmicutes* | | | | | | *Spirochaetes* |
|  | *Bacteroidales* | *Prevotella* | *RF16* | *Paraprevotellaceae* | *CF231* | *YRC22* | *Clostridiales* | *Clostridium* | *Lachnospiraceae* | *Anaerovibrio* | *Selenomonas* | *Mogibacteriaceae* | *Treponema* |
| Total Gas Production, mL/g of DM | 0.420 | 0.313 | 0.150 | -0.339 | 0.469 | -0.140 | -0.234 | 0.491 | -0.426 | 0.096 | 0.251 | 0.538 | -0.405 |
| CH_4_ production, mL/g of DM | **0.723** | 0.423 | 0.297 | *-0.654* | **0.776** | **-0.701** | *-0.603* | **0.731** | *-0.588* | -0.165 | 0.149 | **0.892** | -0.572 |
| H_2,_ mL/g of DM | -0.560 | **-0.891** | *-0.653* | **0.870** | **-0.820** | **0.875** | **0.893** | -0.460 | **0.847** | -0.345 | **-0.706** | -0.431 | **0.887** |
| Total VFA, mM | 0.309 | 0.564 | 0.175 | -0.519 | **0.690** | *-0.616* | -0.418 | 0.217 | *-0.594* | 0.439 | *0.652* | 0.489 | *-0.662* |
| VFA, % molar proportion | |  |  |  |  |  |  |  |  |  |  |  |  |
| Acetate | 0.582 | 0.556 | 0.371 | *-0.657* | **0.766** | **-0.728** | -0.547 | **0.677** | *-0.635* | 0.053 | 0.379 | *0.661* | **-0.667** |
| Propionate | *-0.586* | -0.581 | -0.451 | **0.668** | **-0.758** | **0.723** | 0.550 | **-0.685** | *0.654* | -0.100 | -0.428 | *-0.604* | **0.690** |
| Isobutyrate | -0.084 | -0.076 | 0.421 | 0.082 | -0.158 | -0.078 | 0.118 | 0.104 | 0.112 | 0.006 | 0.003 | -0.367 | 0.074 |
| Butyrate | -0.400 | -0.319 | -0.034 | 0.433 | *-0.583* | 0.556 | 0.390 | -0.492 | 0.390 | 0.132 | -0.094 | **-0.679** | 0.398 |
| Isovalerate | 0.451 | 0.581 | 0.457 | *-0.588* | 0.481 | -0.375 | -0.534 | 0.210 | -0.521 | 0.311 | 0.459 | 0.301 | -0.568 |
| Valerate | **-0.679** | *-0.602* | -0.206 | **0.759** | **-0.867** | **0.797** | *0.652* | *-0.625* | **0.695** | -0.016 | -0.363 | **-0.843** | **0.726** |
| Acetate:Propionate | 0.573 | 0.563 | 0.422 | *-0.652* | **0.753** | **-0.719** | -0.554 | **0.683** | *-0.639* | 0.069 | 0.394 | *0.627* | **-0.668** |
| Ammonia, m*M* | 0.418 | 0.322 | 0.283 | -0.429 | 0.395 | -0.368 | -0.271 | 0.506 | -0.272 | -0.109 | 0.039 | 0.411 | -0.336 |
| pH | -0.332 | 0.033 | 0.173 | 0.192 | -0.079 | 0.096 | -0.196 | -0.268 | 0.037 | 0.169 | 0.028 | 0.007 | 0.131 |

^1^Data presented are Spearman correlation coefficients between bacterial genera with a sequence proportion of ≥ 0.01% and aggregated fermentation parameter responses from control, chloroform, and *A. taxiformis* treatments from set 6 (n=9). Data in bold = (*P* < 0.05); italics = (0.05 < *P* ≤ 0.10).

^2^DM = Dry matter (1.5 g/vessel), VFA = Volatile fatty acid.
